# The expression of miR-513c and miR-3163 was downregulated in tumor tissues compared with normal adjacent tissue of patients with breast cancer

**DOI:** 10.1186/s12920-021-01029-3

**Published:** 2021-07-07

**Authors:** Soheila Delgir, Khandan Ilkhani, Asma Safi, Yazdan Rahmati, Vahid Montazari, Zahra Zaynali-Khasraghi, Farhad Seif, Milad Bastami, Mohammad Reza Alivand

**Affiliations:** 1 Immunology Research Center, Tabriz University of Medical Sciences, Tabriz, Iran; 2 Molecular Genetics, Department of Medical Genetics, Faculty of Medicine, Tabriz University of Medical Sciences, Tabriz, Iran; 3 Department of Thoracic Surgery, Faculty of Medicine, Tabriz University of Medical Sciences/and also Surgery Ward, Nour-Nejat Hospital, Tabriz, Iran; 4 Department of Immunology and Allergy, Academic Center for Education, Culture, and Research, Tehran, Iran

**Keywords:** Breast cancer, Glutamine metabolism, Glutaminase, Fold change, MicroRNA, miR-513c, miR-3163

## Abstract

**Background:**

Breast cancer (BC) is the most invasive cancer with different subtypes that its metabolism is unique compared with normal cells. Glutamine is considered critical nutrition that many cancer cells, particularly BC cells, are dependent on it for growth and proliferation. Therefore, targeting glutamine metabolism, especially enzymes that are related to this pathway, can be beneficial to design anti-cancer agents. Recent evidence has shown that microRNAs (miRNAs), with a short length and single-strand properties, play a prominent role in regulating the genes related to glutamine metabolism, which may control the development of cancer.

**Methods:**

In silico analysis confirmed that miR-513c and miR-3163 might be involved in glutamine metabolism. The expression level of these two miRNAs was evaluated in eighty BC tissues and normal adjacent tissues. Furthermore, GSE38167, GSE38867, GSE42128, GSE45666, and GSE53179 were employed from gene expression omnibus (GEO). The Limma package was utilized to identify differentially expressed miRNAs (DEMs) of mentioned datasets to evaluate miR-513c and miR-3163 expression. Further, in silico analysis was utilized to predict the potential biological processes and molecular pathways of miR-513c and miR-3163, based on their target genes.

**Results:**

In silico studies revealed top categories of biological processes and cellular pathways that might play a critical role in metabolism reprogramming and cancer development and were target genes for miR-513c and miR-3163. The current study showed that miR-513c (*p* value = 0.02062 and FC =  − 2.3801) and miR-3163 (*p* value = 0.02034 and FC =  − 2.3792) were downregulated in tumor tissues compared to normal adjacent tissues. The analysis of GEO microarray datasets showed that miR-513c was downregulated in GSE38167, GSE38867, GSE42128, GSE45666 and GSE53179, whereas there was a significant downregulation of miR-3163 in only two studies, including GSE38867 and GSE42128 that they were in accordance with our experimental results. Furthermore, the subgroup analysis did not show any substantial relationship between expression levels of these two miRNAs and factors such as age, family history of cancer, and abortion history.

**Conclusion:**

MiR-513c and miR-3163 were downregulated in BC tissues, which might serve as tumor suppressors. They are suggested as potential therapeutic targets for patients with BC.

## Background

Breast cancer (BC) is a major health problem among females worldwide [1]. Based on the world health organization (WHO) statistics, this cancer impacts 2.1 million females every year [2], and causes the greatest number of cancer-related mortality among women [3]. In 2018, it was estimated that 98,755 women died from BC that was nearly 15% of all cancer mortality among females worldwide [4]. Moreover, the mean age of the women identified with this disease has been reduced to lower ages in Iran [5]. Breast cancer has been examined from various aspects, but its exact mechanism is still unclear [6]. Various subtypes of BC and their heterogeneity and complexity deteriorate the early detection and treatment of BC [7, 8]. Many studies have suggested that alteration in glutamine metabolism profile may be one of the unique characteristics of BC cells; consequently targeting glutamine metabolism may be important to design anti-cancer strategies against BC development [8–11].

Metabolic reprogramming is a unique event in cancer cell development [12, 13] that is utilized to supply the bioenergetic and biosynthetic demands for accelerating growth and proliferation of cancer cells [14]. In this regard, many cancer cells, especially BC cells, are dependent on glutamine, as the major source of energy and building block for growth and proliferation [15, 16]. Moreover, blocking glutamine pathway can severely influence and suppress cell proliferation [9]. Recently, valuable studies and bioinformatics analysis demonstrated that some emerged microRNAs (miRNAs) can efficiently control glutamine metabolism by targeting critical enzymes, such as glutaminase (GLS), which can provide an opportunity for regulating cancer development [17, 18].

MiRNAs are a class of small non-coding RNAs with 18–25 nucleotides in length, which are post-transcriptionally involved in the regulation of gene expression by degrading targeted mRNAs and/or inhibiting their translation [19–22]. According to bioinformatics and a few experimental analyses, miR-513c and miR-3163 can regulate glutamine metabolism via targeting GLS. Regarding several experimental studies, mir-513c is a tumor suppressor in multiple cancers such as hepatocellular carcinoma [23], prostate cancer [24], glioma [25], neuroblastoma [26], breast cancer [27], and esophageal adenocarcinoma [28], whereas miR-3163 plays vital roles in retinoblastoma cancer stem cell (RCSCs) [29] and non-small cell lung cancer cell (NSCLC) cancers [30]. Therefore, the expression level of these miRNAs can be evaluated to propose potential prognostic markers and their exploitation for control of glutamine metabolism [31]. Since metabolism reprogramming is a common event in cancer cells and this process significantly changes compared with normal cells [32–34], in the current study, the expression levels of both miR-513c and miR-3163 were evaluated in tumor and normal adjacent tissues of patients with BC.

## Methods

### Selection of miRNAs

Based on recent studies, the glutamine metabolic pathways of cancer cells were reviewed to determine the most prominent enzymes. Furthermore, it has been revealed that upregulation of GLS correlate with progression rate and malignancy of cancers, including BC [35]. Accordingly, miRNAs that are involved in this pathway via targeting GLS, were predicted using mirwalk, miRTarBase 7.0, miRDB, and Target Scan Human 7.2 databases. Further, recent experimental studies were reviewed to confirm these miRNAs. Among numerous miRNAs, miR-513c and miR-3163 were selected because these two miRNAs were confirmed in recent studies and dysregulated in multiple cancers, especially mir-513c in BC cell lines. In this regard, we studied the expression level of miR-513c and miR-3163 in tumor and normal adjacent tissues of patients with BC to determine whether these two miRNAs were dysregulated in this condition.

### Clinical specimens

Firstly, 80 samples of tumor and normal adjacent tissues were obtained from patients with BC who underwent surgery at Noor Nejat Hospital, Tabriz. The specimens were provided from mastectomy and lumpectomy in which a portion of the tumor normal adjacent tissue was removed at a distance from the tumor tissue. Thereafter, the spread and invasion of cancer cells were investigated by pathological examination. In addition, tumor margin samples that were considered healthy by a pathologist were examined as the controls. Written informed consent was obtained from all the participants after explaining the study. The study was confirmed by the Ethics Committee (Ethics code: IR.TBZMED.REC.1398.025) of Tabriz University of Medical Sciences, Tabriz, Iran. Patients did not receive chemotherapy or radiotherapy before the surgery. The specimens were collected after surgical resection, were immediately frozen, and stored at − 80 °C. Clinical pathological features are shown in Table 1.

**Table 1:** Basis characteristics of the patients with breast cancer

Parameter	Number (percentage)
Tumor grade	Grade 3 = 5%
	Grade 2 = 87.5%
	Grade 1 = 7.5%
Lymph node	Yes = 87.5%
	No = 12.5%
Family cancer history	Yes = 47.5%
	No = 41.3%
	Unknown = 11.2%
Abortion history	Yes = 36.2%
	No = 51.3%
	Unknown = 12.5%
Age	≤ 50 38.8%
	> 50 40%
	Unknown = 21.2%

### Total RNA extraction

For extraction of total RNA, the tissue samples were homogenized with liquid nitrogen and their RNA was extracted by Trizol reagent (Geneall). Then, the quality and quantity of extracted RNAs were assessed using a NanoDrop spectrometer (Thermo Scientific, USA). After extraction, obtained RNAs were eluted in 50 μL of RNase-free water and stored at − 80 °C.

### cDNA synthesis and real-time PCR

In this study, cDNA of the miR-3163, miR-513c, and RNU6 were synthesized using reverse transcriptase enzyme (Thermo Fisher, USA), dNTP (Cinnaclon, Iran), and their unique stem-loop-primers. For this purpose, three specific stem-loop primers were designed for miR-3163, miR-513c, and also RNU6 (for normalization) were employed and the conditions of PCR machines were 30 min at 16 °C, 30 min at 42 °C, and 5 min at 75 °C for conducting Real-time PCR reaction, SYBR Green master mix (Amplicon, Denmark), miR-3163, miR-513c specific primers, and dNTP were used. These reactions were performed by MIC PCR bimolecular system in two steps as follows: for miR-513c: 10 min at 94 °C, 40 cycles of 15 s at 94 °C and 30 s at 58 °C. For miR-3163: 10 min at 94 °C, 40 cycles of 15 s at 94 °C and 20 s at 57 °C. Then, for RNU6 amplification: 10 min at 94 °C, 40 cycles in 15 s at 94 °C and 20 s at 56 °C. The results were shown by mic-PCR v1.4.0 software. The sequences of the primers are shown in Table 2.

**Table 2:** cDNA synthesis specific stem-loop primers and real-time PCR primer sequences

	Noncoding-RNAs and their accession numbers	Sequences	
cDNA synthesis reaction	MiR-3163-5p NIMAT 0015037	MiR-3163 (STL)	5′-GTCGTATCCAGTGCAGGGTCCGAGGTATTCGCACTGGATACGACGTCTTAC-3′
	Mir-513c-5p NIMAT 0022728	Mir-513c (STL)	5′-GTCGTATCCAGTGCAGGGTCCGAGGTATTCGCACTGGATACGACATAAAC-3′
	RNU6 NR_003027.2	U6 (STL)	5′-GTCGTATCCAGTGCAGGGTCCGAGGTATTCGCACTGGATACGACAAAAATAT-3′
Real time PCR reaction	MiR-3163-5p NIMAT 0015037	Mir-3163 (F)	5′-AGGGTATAAAATGAGGGCAGTAAGAC-3′
	Mir-513c-5p NIMAT 0022728	Mir-513c (F)	5′-GGGTTCTCAAGGAGGTGTCG-3′
	MiR-3163 and Mir-513c	Common (R)	5′-GTGCAGGGTCCGAGGT-3′
	RNU6 NR_003027.2	U6 (F)	5′-GCTTCGGCAGCACATATACTAAAAT-3′
		U6 (R)	5′-CGCTTCACGAATTTGCGTGTCAT-3′

### In silico analysis

Firstly, we selected and evaluated some microRNA microarray datasets of BC cancer studies from GEO (Gene Expression Omnibus) to confirm miR-3163 and miR-513c expression. In this regard, we downloaded the expression profiling array data of GSE38167 (67 samples, including 31 primary TNBC, 13 lymph node metastases BC, and 23 matched normal breast tissues), GSE38867 (28 samples, including 7 DCIS BC, 7 invasive BC, 7 metastatic BC, and 7 normal samples), GSE42128 ( 116 samples, including 68 serum samples, 28 BC tissue samples and 20 normal breast tissue samples), GSE45666 (116 samples, including 101 breast tumor samples and 15 from adjacent breast normal tissue samples) and GSE53179 (16 blood samples, including 11 samples from ER+/HER2− advanced breast cancer subjects and 5 age-matched control subjects) from GEO database (https://www.ncbi.nlm.nih.gov/geo/). Then, we normalized the expression array using Quantile Normalization function in Limma package [36]. Using the aggregate function in the S4 Vectors package, which gives an average measure for the probes of each miRNA. The detection of differentially expressed miRNAs (DEMs) between BC and healthy samples was performed Limma package (cut-off: $\left|{\mathrm{logFC}}\right|$ > 0.5 and *p* value < 0.05). Then, we performed a survival analysis to evaluate their prognostic performance of miR-513c-5p and miR-3163 based upon mentioned microarray data in BC. Furthermore, we performed a survival analysis to evaluate their prognostic performance of miR-513c-5p and miR-3163 based upon mentioned microarray data in BC.

Further, In silico analysis was performed to evaluate biological processes and cellular pathways that were enriched by targeted genes of miR-513c-5p and miR-3163. The experimentally confirmed target genes of miR-513c-5p and miR-3163 were obtained from the miRTarBase 6.0 database (http://mirtarbase.mbc.nctu.edu.tw/php/index.php) and exposed to overrepresentation enrichment analysis (ORA) based on the Gene Ontology (GO) and the Kyoto Encyclopedia of Genes and Genomes pathways using the Web Gestalt (http://www.webgestalt.org/option.php) webserver. In both analyses, the reference gene list and the multiple test adjustment method were set to “genome_protein_coding” and Benjamini–Hochberg, respectively. In the GO analysis, the top important categories were selected. Other parameters were set as defaults.

### Statistical analysis

All experiments were performed at least two times*.* Shapiro–Wilk test was checked whether data were normally distributed. Paired T-test and delta-CTs were used for comparison of the gene expressions between tumor and normal adjacent tissues. Two- sample T-test and logarithm2 of FC were applied to compare expression alterations of genes in tumors compared to normal adjacent tissue across subsets of study samples*.* All statistical analyses were conducted using R software version 0.5. 1. Finally, *p* < 0.05 was considered statistically significant. Receiver operating characteristics (ROC) curve analysis was performed to evaluate potential biomarker in these miRNAs.

## Results

Based on miRTarBase, miRDB, and Target Scan Human 7.2, as miR- related databases, miR-513c and miR-3163 were selected as potential miRNAs that might play a significant role in the regulation of glutamine metabolism pathway via targeting GLS. Based on recent experimental studies, miR-513c has tumor-suppressive roles, while miR-3163 plays diverse roles in several cancers [27, 29, 37]. To determine the roles of miR-513c and miR-3163 in human BC, we compared the expressions of both miRNAs in BC tissues and their normal adjacent tissues.

### MiR-513c and miR-3163 were downregulated in BC tissues

#### MiR-513c

**Fig. 1:**
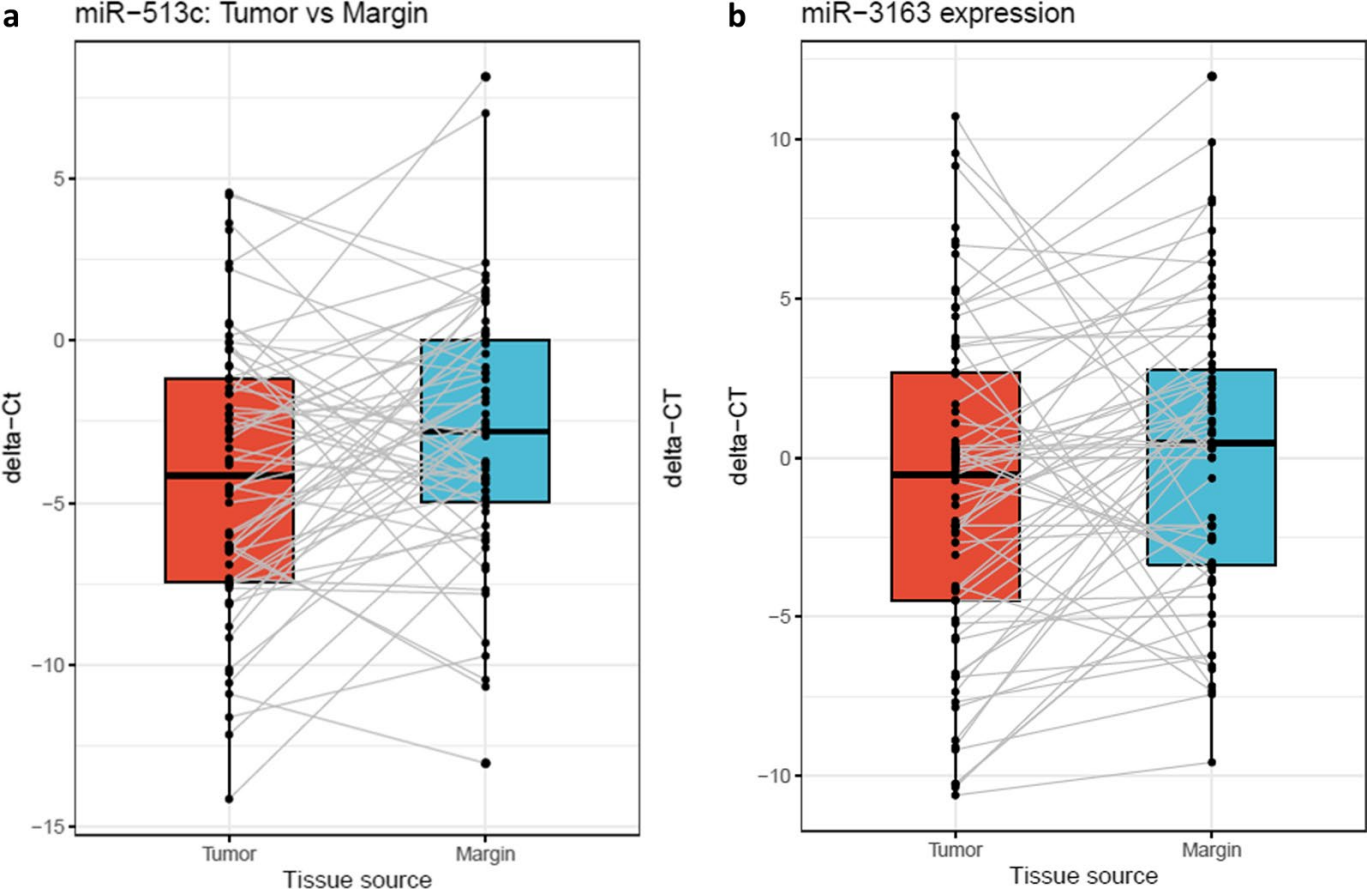
The expression of miRNAs in tumor tissues and normal adjacent tissues. **a** The expression of miR-513c in tumor tissues and normal adjacent tissues. **b** The expression of miR-3163 in tumor tissues and normal adjacent tissues. In both figures, vertical axis, center line, and error bars represent ΔCTs values, median, and interquartile range, respectively

The results of paired T-test showed that the expression level of miR-513c was significantly downregulated in the human BC tissues compared to the normal adjacent tissues (*p* value = 0.02062) and (FC =  − 2.3801) (Fig. 1a).

#### MiR-3163

The results of paired T-test revealed that the expression level of miR-3163 was significantly downregulated in the human BC tissues compared to the normal adjacent tissues (*p* value = 0.02034) and (FC =  − 2.3792) (Fig. 1b).

#### Receiver operating characteristics (RUC) curve analysis of MiR-513c and MiR-3163

**Fig. 2:**
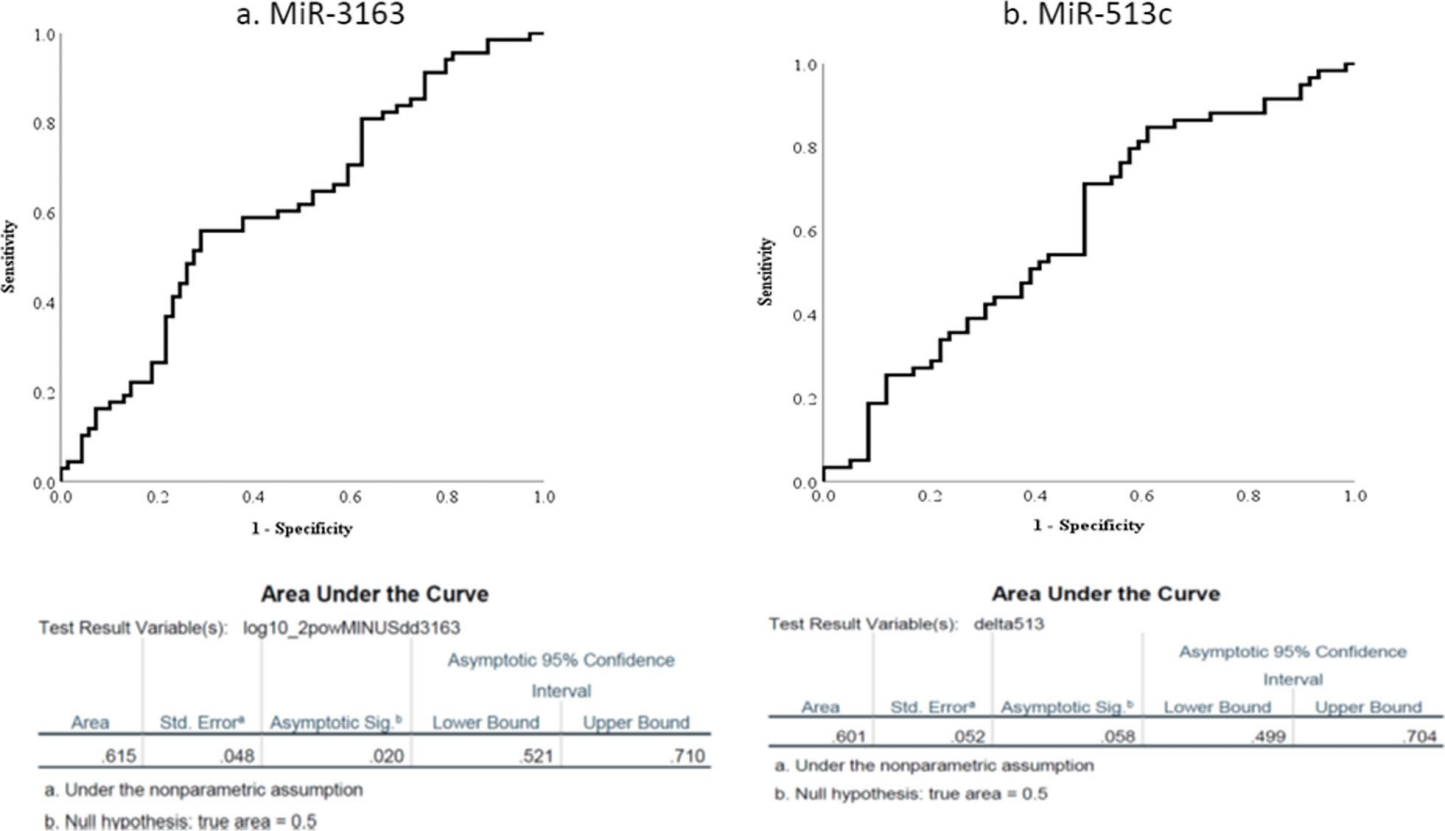
The curve analysis of both MiR-3163 (**a**) and MiR-513c (**b**) with AUCs (0.61 and 0.60, respectively)

The curve analysis of both MiR-513c and MiR-3163 showed that AUC (area under the curve) of MiR-513c and MiR-3163 are 0.61 and 0.60 in these samples, respectively; suggesting that both of them could be considered in patients with BC (Fig. 2a, b).

### Subgroups analysis

In this study, the mean ± SD of age, age of first menstruation, age of first pregnancy and BMI were 51.28 ± 12.5, 12.28 ± 2.7, 22.41 ± 6.22 and 29.22 ± 5.49, respectively. Additionally, there was no significant difference in the expression level of both MiR-3163 and miR-513c between over 50 and under 50 years, and pre-menopause and post-menopause subjects. Although the expression level of miR-513c was significantly different (*p* = 0.007) between tumor and normal tissues in patients with grade 2, no significant difference was observed in terms of MiR-3163 (*p* = 0.21). In addition, there was no significant difference in the expression level of both miRNAs in patients with grade 1 and 3 (*p* > 0.05). To show the relationship between the expression of miR-513c and miR-3163 with factors such as age, family history cancer, and abortion in BC subgroups, two-sample T-test of logarithm2 of fold changes were employed.

#### MiR-513c

**Fig. 3:**
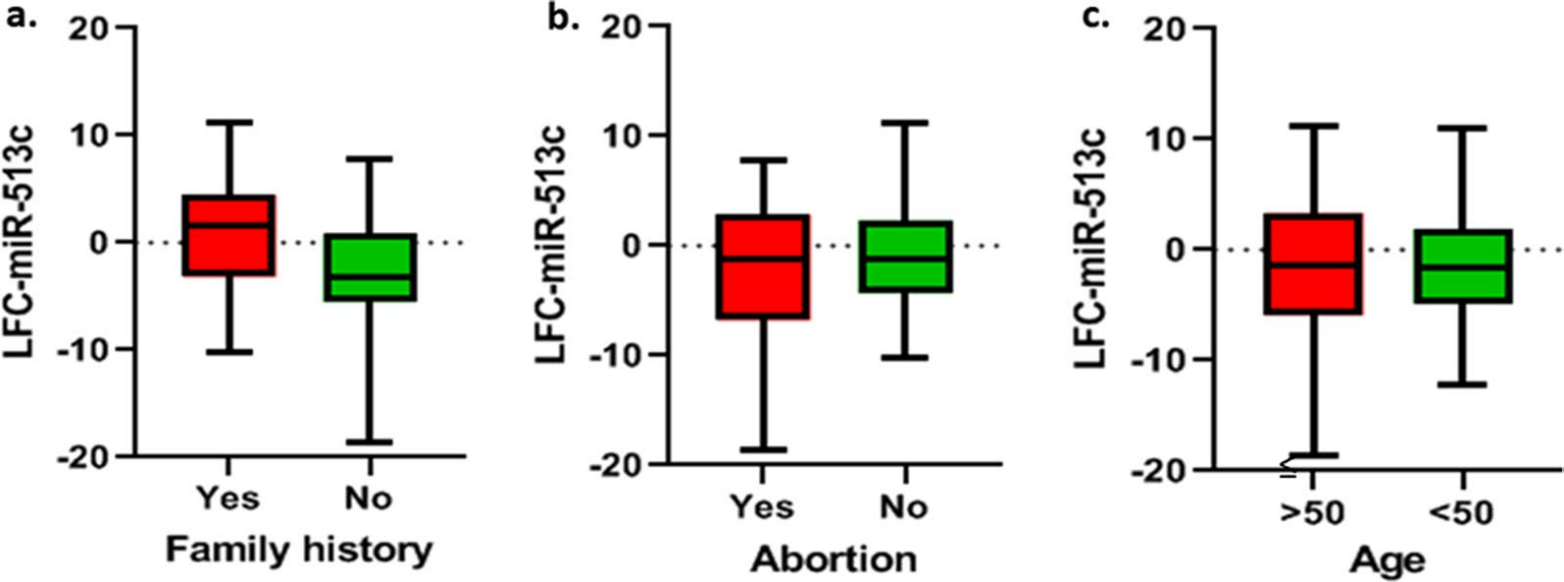
The correlation of miR-513c expression level with age, abortion history, and cancer family history. **a** LFC-miR-513c in patients with cancer family history and without cancer family history (*p* value = 0.0525). **b** LFC-miR-513c in patients with abortion history and without abortion history (*p* value = 0.7713). **c** LFC-miR-513c in patients with age ≥ 50 and < 50 (*p* value = 0.6758). In three figures, vertical axis, center line, and error bars represent LFC (i.e., base 2 logarithm of FC), median, and interquartile range, respectively

We did not find any significant changes in the expression level of miR-513c in patients with cancer family history and without cancer family history (*p* value = 0.0525, F C =  − 1.9859) (Fig. 3a). Additionally, there was no significant relationship between the expression of miR-513c with abortion history (*p* value = 0.7713, FC = 0.29275) (Fig. 3b) and age subgroups of ≤ 50 years and > 50 years (*p* value = 0.6758, FC =  − 0.42103) (Fig. 3c).

#### MiR-3163

**Fig. 4:**
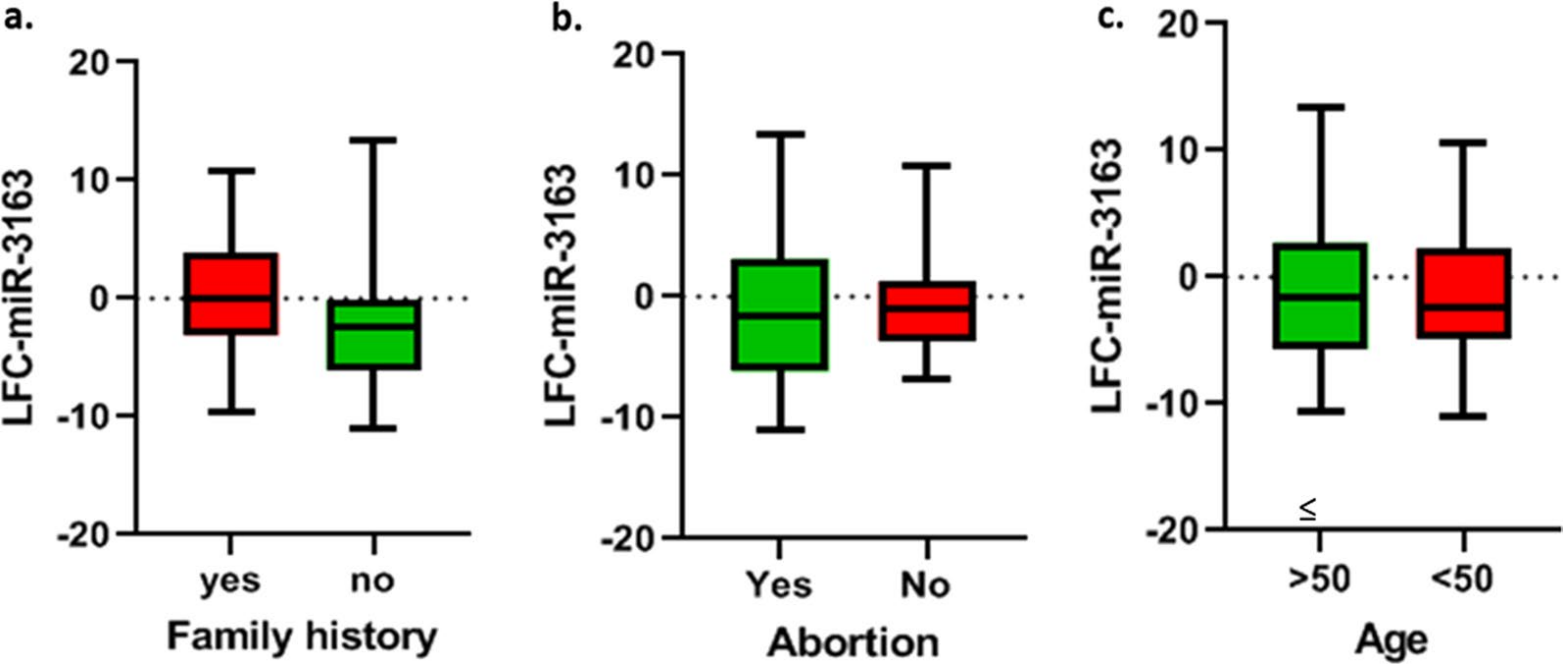
The correlation of miR-3163 expression level with age, abortion history, and cancer family history. **a** LFC-miR-3163 in patients with cancer family history and without cancer family history (*p* value = 0.1208). **b** LFC-miR-3163 in patients with abortion history and without abortion history (*p* value = 0.5634). **c** LFC-miR-3163 in patients with age < 50 and ≥ 50 (*p* value = 0.3805). In three figures, vertical axis, center line, and error bars represent LFC (i.e., base 2 logarithm of FC), median, and interquartile range, respectively

Based on results of paired sample t-test, there was no markedly changes in the expression level of miR-3163 in subgroups of cancer family history (*p* value = 0.1208, FC =  − 1.5756) (Fig. 4a). Additionally, there was no significant relationship between the expression of miR-3163 with abortion history (*p* value = 0.5634, FC = 0.58281) (Fig. 4b), and age in patients ≤ 50 and > 50 (*p* value = 0.3805, FC = 0.8859) (Fig. 4c).

### In silico analysis

**Fig. 5:**
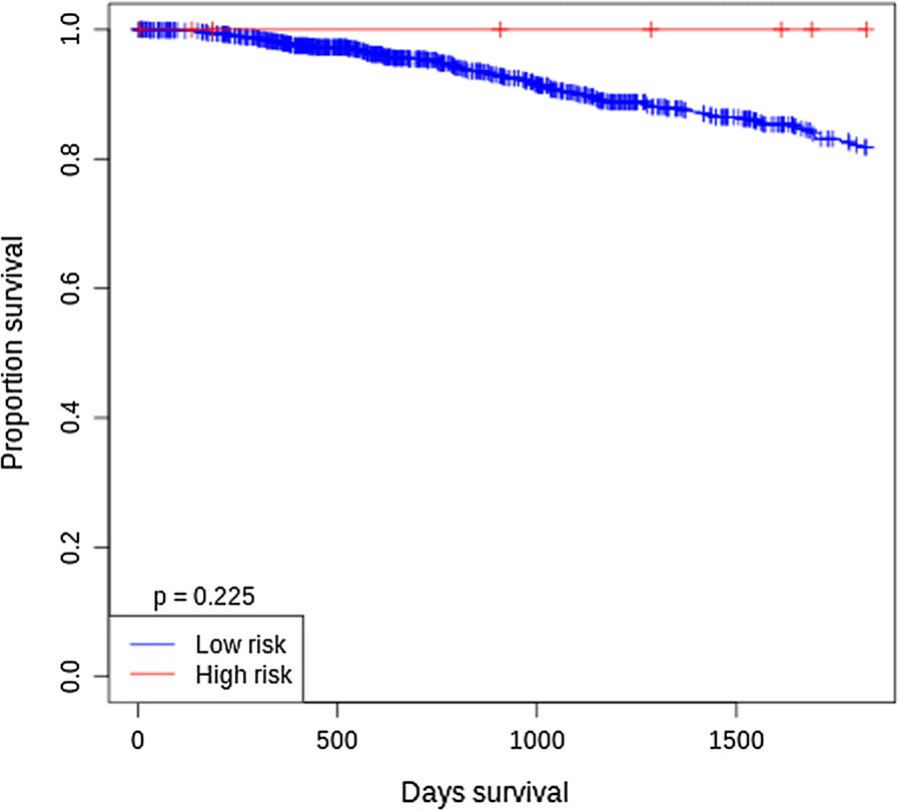
Overall survival analysis of miR-513c-5p in BC from related datasets

**Fig. 6:**
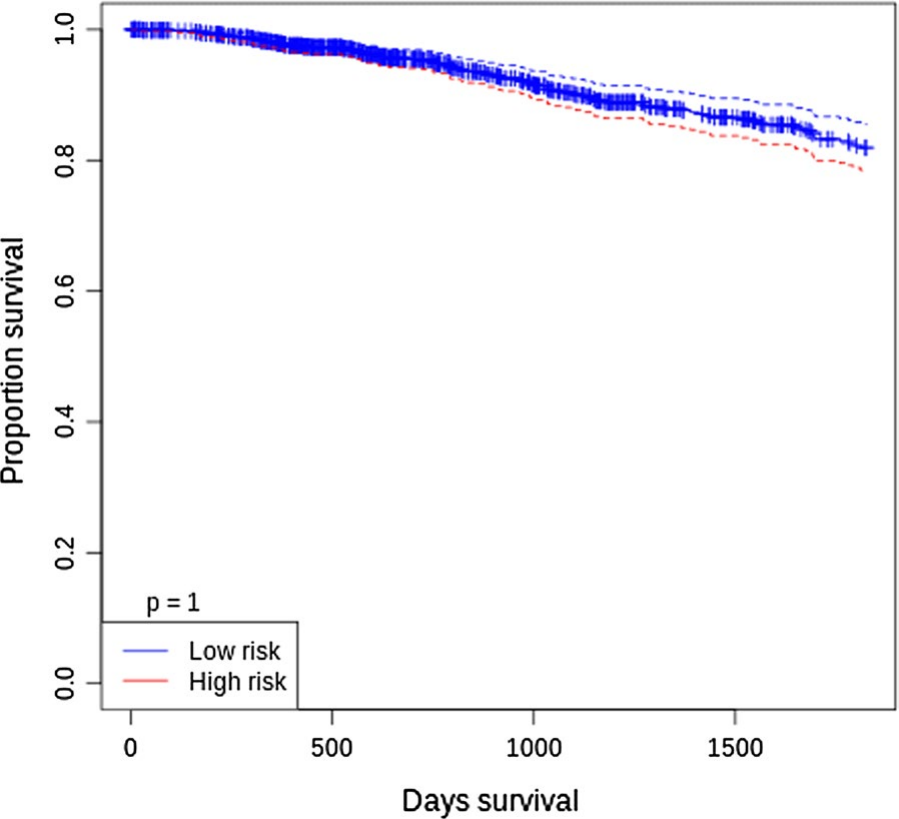
Overall survival analysis of miR-3163 in BC from related datasets

Considering |log2FC| > 0.5 and *p* < 0.05 as the cut-off, we detected that miR-513c in GSE38167 ($log FC = - 1.00284$), GSE38867 ($logFC=-1.4$), GSE42128 ($logFC=-3.1$), GSE45666 ($logFC=-1.8$), and GSE53179 ($logFC=-0.9$) were downregulated, whereas there was a significant miR-3163 downregulation in only two array datasets, GSE38867 ($logFC$= − 0.6) and GSE42128 ($logFC=$ − 2.4) that they were in accordance with our experimental results. According to survival plots related to miR-513c-5p and miR-3163 in BC showed that prognostic performance of miR-3163 was higher than miR-513c-5p and therefore, according to array data, the prognostic potency of miR-3163 was higher for assessment of BC risk (Figs. 5, 6).

**Fig. 7:**
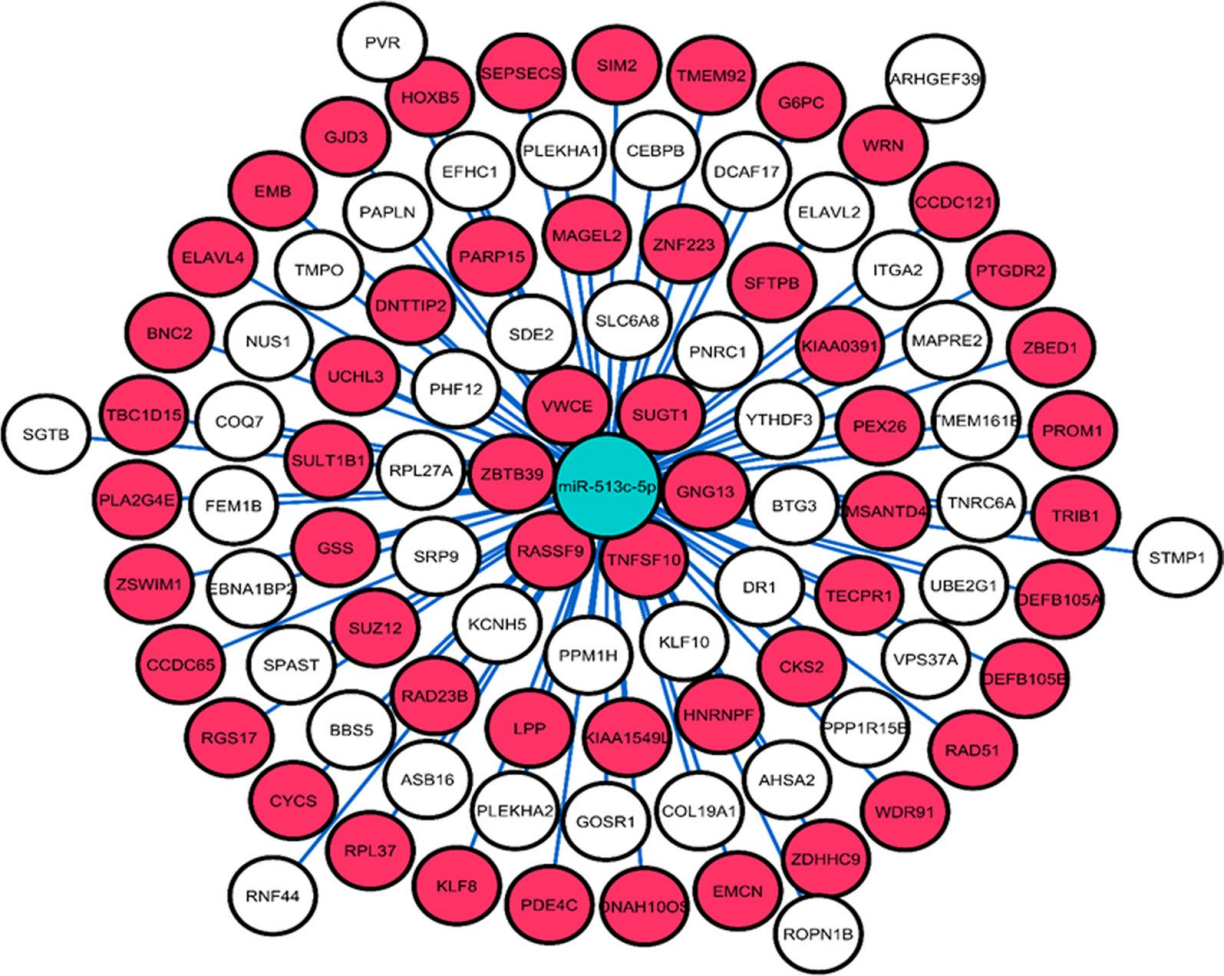
The interaction between miR-513c-5p and its target genes

**Fig. 8:**
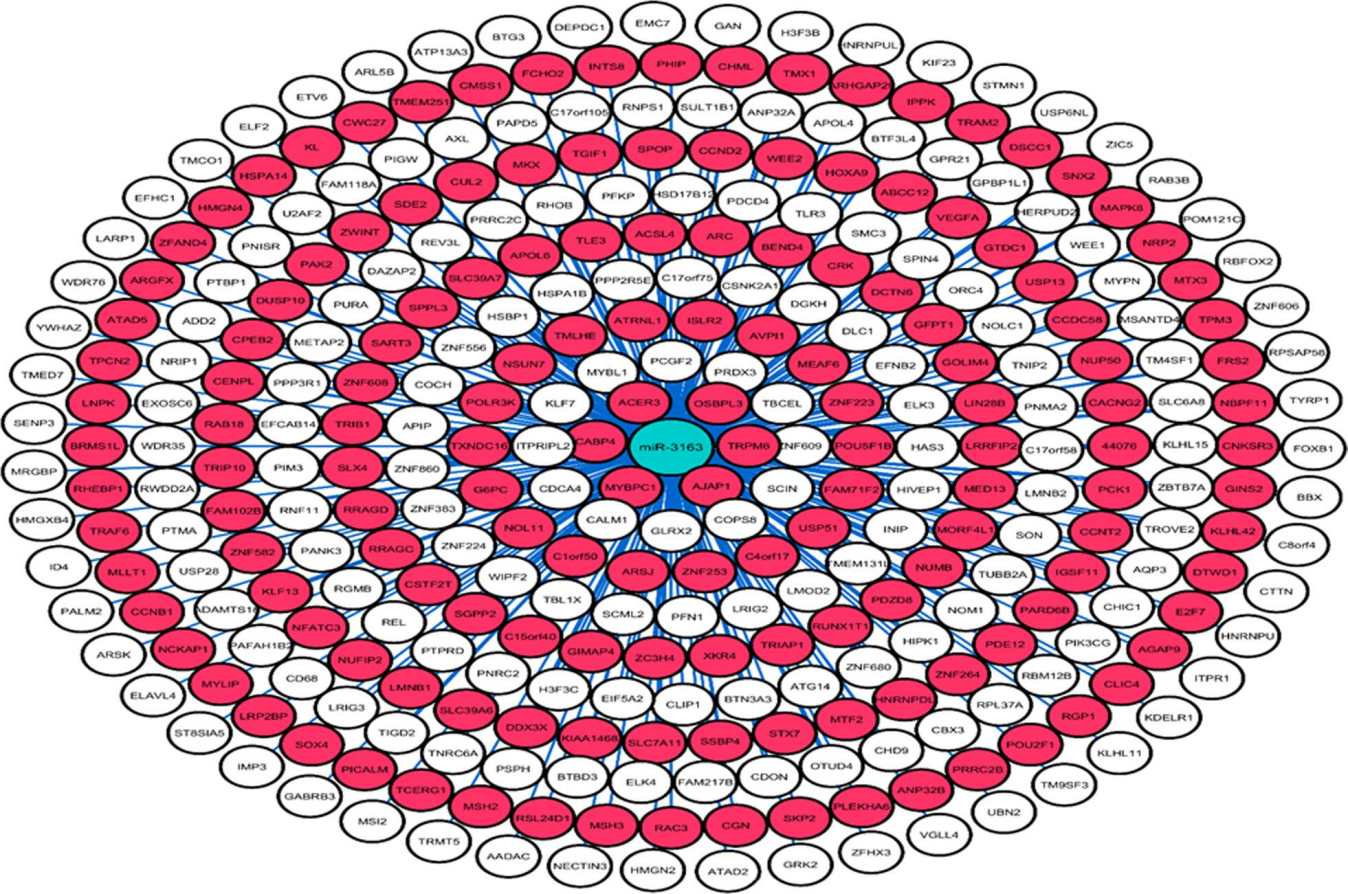
The interaction between miR-3163 and its target genes

A total of 98 and 333 experimentally confirmed target genes for miR-513c and miR-3163 were extracted from the miRTarBase database, respectively. Cytoscape V 3.7.1 (Figs. 7, 8) portrayed the miRNA-gene interaction. The ORA showed that significant categories of biological procedures were overrepresented among target genes of miR-513c and miR-3163 (Tables 3, 4). Pathway analysis revealed top biological enriched pathways and processes in target genes of miR-513c and miR-3163 (Tables 5, 6). Based on these analyses, target genes of miR-513c are involved in pathways related to cancer development. Furthermore, substantial biological processes were enriched among target genes of miR-3163, such as negative regulation of nucleobase-containing compound metabolic process, regulation of mRNA metabolic process, positive regulation of nucleobase-containing compound metabolic process, negative regulation of RNA metabolic process, and negative regulation of cellular macromolecule biosynthetic process, are associated with metabolism pathways. Also, MAPK, Hedgehog, and Wnt signaling pathways were the most prominent pathways among several cellular pathways that were considerably influenced by miR-3163. These biological processes and cellular pathways were related to BC progression and cancer metabolism.

**Table 3:** Pathways that are enriched among target genes of mir-513c

No	ID	Pathway name	O	FDR	E	*p* value^a^
1	hsa05222	Small cell lung cancer	92	1	0.34163	0.0046026
2	hsa00130	Ubiquinone and other terpenoid-quinone biosynthesis	11	1	0.040847	0.040124
3	hsa05200	Pathways in cancer	524	1	1.9458	0.041062
4	hsa05032	Morphine addiction	91	1	0.33792	0.044411

*E* number of expected genes in each category, *O* number of observed target genes in each specific category

^a^The Benjamini–Hochberg adjusted *p* value

**Table 4:** Pathways that are enriched among target genes of miRNA-3163

Nos	Gene set	Pathway name	C	E	R	FDR	*p* value^a^
1	hsa05170	Human immunodeficiency virus 1 infection	212	3.6155	3.3191	0.081094	2.5E−04
2	hsa04110	Cell cycle	124	2.1147	3.783	0.09676	1E−03
3	hsa04114	Oocyte meiosis	124	2.1147	3.783	0.09676	1E−03
4	hsa05202	Transcriptional misregulation in cancer	186	3.1721	3.1525	0.09676	1E−03
5	hsa04010	MAPK signaling pathway	295	5.0309	2.584	0.09676	1.5E−03
6	hsa05167	Kaposi sarcoma-associated herpesvirus infection	186	3.1721	2.8373	0.2378	4.4E−03
7	hsa04340	Hedgehog signaling pathway	47	0.80154	4.9904	0.3802	8.2E−03
8	hsa04310	Wnt signaling pathway	146	2.4899	2.8114	0.49589	1E−02
9	hsa05206	MicroRNAs in cancer	150	2.5581	2.7364	0.50585	1.4E−02
10	hsa04218	Cellular senescence	160	2.7286	2.5654	0.58131	2E−02

*C* the total number of genes in the category, *E* number of expected genes in each category, *R* fold enrichment

^a^The Benjamini–Hochberg adjusted *p* value

**Table 5:** Top ten enriched biological processes among target genes of miR-513c

No	GO ID	Biological process	O	FDR	E	R	*p* value^a^
1	GO: 0042148	Strand invasion	5	1	0.024004	83.32	2.2E−04
2	GO: 0006612	Protein targeting to membrane	187	1	0.89774	6.6834	2.7E−04
3	GO: 0045047	Protein targeting to ER	107	1	0.51368	7.7869	1.7E−03
4	GO: 0072599	Establishment of protein localization to endoplasmic reticulum	111	1	0.53289	7.5063	1.9E−03
5	GO: 0008340	Determination of adult lifespan	17	1	0.081613	24.506	2.9−E3
6	GO: 0006605	Protein targeting	412	1	1.9779	3.5391	3.6E−03
7	GO: 0090150	Establishment of protein localization to membrane	313	1	1.5026	3.993	3.9–03
8	GO: 0070972	Protein localization to endoplasmic reticulum	137	1	0.65771	6.0818	4.2E−03
9	GO: 0031297	Replication fork processing	95	1	0.15362	13.019	1E−02
10	GO: 0006614	SRP-dependent co-translational protein targeting to membrane	95	1	0.45607	6.5779	1E−02

*C* the total number of genes in the category, *E* number of expected genes in the category, *GO* gene ontology, *R* fold enrichment, *O* number of observed target genes in each specific category

^a^The Benjamini–Hochberg adjusted *p* value

**Table 6:** Top ten enriched biological processes among target genes of miR-3163

No	Gene set	Biological pathways	C	E	R	FDR	*p* value
1	GO: 0045934	Negative regulation of nucleobase-containing compound metabolic process	1418	22.805	1.9732	0.04352	8E−06
2	GO: 0033554	Cellular response to stress	1867	30.026	1.7984	0.04352	1E−05
3	GO: 0010629	Negative regulation of gene expression	1733	27.871	1.8299	0.04352	1E−05
4	GO: 1903311	Regulation of mRNA metabolic process	266	4.278	3.5063	0.06326	3E−05
5	GO: 0045935	Positive regulation of nucleobase-containing compound metabolic process	1847	29.705	1.7169	0.12664	8E−05
6	GO: 0000278	Mitotic cell cycle	927	14.909	2.0793	0.12664	9E−05
7	GO: 0051253	Negative regulation of RNA metabolic process	1295	20.827	1.8726	0.12664	1E−04
8	GO: 0000902	Cell morphogenesis	982	15.793	2.0262	0.12664	1E−04
9	GO: 0032989	Cellular component morphogenesis	1082	17.401	1.9539	0.12664	1E−04
10	GO: 2000113	Negative regulation of cellular macromolecule biosynthetic process	1359	21.856	1.8301	0.12664	1E−04

## Discussion

MiRNAs have been demonstrated as promising biomarkers for diagnosis and prognosis of BC [38, 39]. Irregular expression of miRNAs impacts the processes involved in BC development such as invasion, metastasis, promoting tissue, stimulating anti-apoptotic activity, drug resistance, and metabolism reprogramming [19, 40, 41]. These critical molecules have been found as key players in cancer metabolism by regulating genes related to metabolism pathways [42]. According to bioinformatics databases, miRNAs such as miR-513c and miR-3163 involved in glutamine metabolism were evaluated in the present study. Our findings demonstrated that mir-513c and miR-3163 were significantly downregulated in BC tissues compared to normal adjacent tissues.

To the best of our knowledge, tumor suppressor role of miR-513c in multiple cancers has been established. In R2N1d and MDA-MB-231 BC cell lines, considering treatment with Histone deacetylase inhibitors (HDACi), among the most significantly expressed miRNAs, miR-513c also emerged as the most upregulated gene, and acts as a tumor suppressor in the induction of cell death [27]. Furthermore, miR-513c is markedly downregulated in hepatocellular carcinoma and glioblastoma (GBM) and overexpression of this miRNA prevented the proliferation of these cancer cells through targeting MET and Wnt/β-catenin signaling pathway, respectively [23, 25]. Additionally, miR-513c is markedly downregulated in neuroblastoma. Moreover, miR-513c, as a tumor suppressor, plays an important role in regulating glutamine metabolism by targeting GLS [26].

Since the role of miR-513c-5p has not been fully understood, bioinformatics analysis was done to shed the light on molecular pathways and biological procedures that are potentially impacted by dysregulation of miR-513c-5p*.* In silico studies indicated that target genes of miR-513c are involved in pathways related to cancer progression. In the present study, miR-513c was downregulated in BC tissues compared to normal adjacent tissues (*p* value = 0.02062, FC =  − 2.3801). Therefore, it seems miR-513c may serve as a tumor suppressor and may have a considerable role in the development of BC.

In previous studies, the activity and expression of miR-3163 have been evaluated in multiple cancers such as NSCLC and Retinoblastoma (RB). In addition, miR-3163 as a moderator contributes to Meg3 to suppress and regulate the translation of Skp2 in NSCLC. However, miR-3163 expression does not differ in NSCLC compared with normal cells, which suppresses the Skp2 translation and reduction of its level in combination with Meg3 to decrease cell proliferation in NSCLC [30]. Also, the upregulation of miR-3163 via targeting ABCG2 reduced the multidrug resistance and promotion of apoptosis in RCSC [29].

Because the activity of miR-3163 has not been completely understood, bioinformatics analyses were performed to determine a significant relationship between cancer and miR-3163. This analysis provides an insight into molecular pathways and biological processes that are potentially regulated by target genes of miR-3163. Based on this analysis, among important biological processes, negative regulation of cellular macromolecule biosynthetic process (GO: 2000113) is associated with metabolism pathways [43]. Also, significant cellular pathways enriched by miR-3163 are related to cancer cell progression, such as MAPK, Hedgehog, and Wnt signaling pathways are the most important. MAPK and Hedgehog signaling pathways are critical key regulators in cellular functions such as cell differentiation, proliferation, differentiation, survival, and apoptosis. The higher activation of MAPK in subtypes of BC predicts invasive phenotypes and poor prognosis [44–46]. Furthermore, these signaling pathways can also affect metabolic pathways by directly regulating the expression of critical enzymes in metabolic pathways [47–49]. Also, Wnt signaling pathway, as a master regulator, plays a significant role in the progression and development of BC by metabolism reprogramming, including glutamine metabolism pathway [50, 51]. So, this information may provide a valuable clue about cancer metabolism and our preliminary theory.

Additionally, the GEO microarray datasets analysis showed that both miR-513c and miR-3163 were downregulated in GSE38867 and GSE42128 datasets. Other analyzed array datasets such as GSE38167, GSE45666, and GSE53179 were significant only for downregulated miR-513c. According to survival plots of miR-513c and miR-3163 in BC, it has been demonstrated that miR-3163 may be a potent prognostic risk factor in BC (Figs. 5, 6).

The findings of the present study, contrary to previous studies, demonstrated that the expression of mi-3163 was downregulated in BC tissues compared with normal adjacent tissues (*p* value = 0.02034, FC =  − 2.3792). Recent reports have shown that miR-3163 plays diverse roles in various cancers; nonetheless, further studies are needed to identify and validate the precise role of this miRNA. According to our results and bioinformatics analyses such as the related data sets, it seems that miR-3163 may play an outstanding role in BC development.

## Conclusion

Altogether, mir-513c and mir-3163 expression were downregulated in BC tissues in comparison to normal adjacent tissues. Therefore, it seems that mir-513c and mir-3163 may serve as tumor suppressors in patients with BC. Furthermore, no significant association was found between miR-513c and miR-3163 expression and variables such as age, cancer family history, and abortion. Although these miRNAs may be suggested as therapeutic targets, further studies are needed to elucidate molecular mechanisms and validate the predicted findings using bioinformatics studies.

## Data Availability

The data that support the findings of this study are available on request from the corresponding author. The data are not publicly available due to privacy or ethical restrictions e.g. containing information that could compromise the privacy of research participants.

## Abbreviations

BC: Breast cancer
GLS: Glutaminase
HCC: Hepatocellular carcinoma
LPR6: Low-density lipoprotein (LDL) receptor-related protein-6
RB: Retinoblastoma
GBM: Glioblastoma
ABCG2: ATP binding cassette subfamily G member 2
MEG3: Maternally expressed 3
LFC: Logarithm fold change
RCSCs: Retinoblastoma cancer stem cells
NSCLC: Non-small cell lung cancer
HDACi: Histone deacetylase inhibitor
WHO: World Health Organization
